# Dynamics of MDR *Enterobacter cloacae* outbreaks in a neonatal unit in Nepal: insights using wider sampling frames and next-generation sequencing

**DOI:** 10.1093/jac/dku521

**Published:** 2015-01-03

**Authors:** N. Stoesser, A. E. Sheppard, M. Shakya, B. Sthapit, S. Thorson, A. Giess, D. Kelly, A. J. Pollard, T. E. A. Peto, A. S. Walker, D. W. Crook

**Affiliations:** 1 Nuffield Department of Clinical Medicine, University of Oxford, Oxford, UK; 2 NIHR Biomedical Research Centre, University of Oxford/Oxford University Hospitals NHS Trust, Oxford, UK; 3 Department of Paediatrics, Patan Hospital, Kathmandu, Nepal; 4 Oxford Vaccine Group, Centre for Clinical Vaccinology and Tropical Medicine, University of Oxford, Oxford, UK

**Keywords:** NDM, carbapenemases, whole-genome sequencing, epidemiology

## Abstract

**Objectives:**

There are limited data on *Enterobacter cloacae* outbreaks and fewer describing these in association with NDM-1. With whole-genome sequencing, we tested the hypothesis that a cluster of 16 *E. cloacae* bacteraemia cases in a Nepali neonatal unit represented a single clonal outbreak, using a wider set of epidemiologically unrelated clinical *E. cloacae* isolates for comparison.

**Methods:**

Forty-three isolates were analysed, including 23 *E. cloacae* and 3 *Citrobacter* sp. isolates obtained from blood cultures from 16 neonates over a 3 month period. These were compared with two contemporaneous community-associated drug-resistant isolates from adults, a unit soap dispenser isolate and a set of historical invasive isolates (*n* = 14) from the same geographical locality.

**Results:**

There were two clear neonatal outbreaks and one isolated case in the unit. One outbreak was associated with an NDM-1 plasmid also identified in a historical community-associated strain. The smaller, second outbreak was likely associated with a contaminated soap dispenser. The two community-acquired adult cases and three sets of historical hospital-associated neonatal isolates represented four additional genetic clusters.

**Conclusions:**

*E. cloacae* infections in this context represent several different transmission networks, operating at the community/hospital and host strain/plasmid levels. Wide sampling frames and high-resolution typing methods are needed to describe the complex molecular epidemiology of *E. cloacae* outbreaks, which is not appropriately reflected by routine susceptibility phenotypes. Soap dispensers may represent a reservoir for *E. cloacae* and bacterial strains and plasmids may persist in hospitals and in the community for long periods, sporadically being involved in outbreaks of disease.

## Introduction

*Enterobacter cloacae* can colonize the human gastrointestinal tract and is an emerging drug-resistant nosocomial pathogen,^1^ particularly in neonatal critical care.^2,3^ Several previous outbreaks in neonatal units have been attributed to a variety of sources such as contaminated medications, distilled water in mechanical ventilators and solutions used for parenteral nutrition. These are typically controlled by review and improvement of infection control practices.^4^ When molecular typing has been deployed as part of epidemiological investigation, most outbreaks appear clonal, based predominantly on analysis by PFGE.^4^

The New Delhi metallo-β-lactamase (NDM) carbapenemase was first identified in a *Klebsiella pneumoniae* strain in a patient originally hospitalized in India.^5^ Since then, ≥11 different gene variants have emerged globally,^6^ in several different plasmid backgrounds and bacterial species.^7^ South Asia is one potential reservoir for these genes, with an NDM carriage prevalence of 14% in outpatients and 27% in hospitalized patients reported in Pakistan^8^ and NDM-1 genes detected in 4% of drinking water and 30% of waste seepage samples from New Delhi, India.^9^ Most published descriptions of clinical outbreaks have been associated with NDM-positive *K. pneumoniae* or *Escherichia coli*^10,11^ and, again, large outbreaks are typically thought to be clonal using a variety of typing methodologies, including repetitive-PCR or high-resolution typing with whole-genome sequencing (WGS).^12^

*E. cloacae* outbreaks in association with *bla*_NDM_ have also been observed in epidemiological surveys,^13,14^ but the specific dynamics of an outbreak caused by NDM-producing *E. cloacae* have not yet been defined. In this study, we used WGS to test the hypothesis that a set of *E. cloacae* isolates, including carbapenem-resistant strains, collected from a temporally associated cluster of cases in a neonatal unit in Nepal, represented a single clonal outbreak.

## Methods

### Hospital setting

The study took place in a 450 bed general hospital in Kathmandu, which handles ∼9500 live births/year; ∼7000 neonates are admitted after delivery. Neonatal care is managed in two nurseries: one ‘clean’, for non-infection-related supportive care, and one ‘septic’, for patients who have a likely diagnosis of/risk factors for sepsis. The neonatal ICU (NICU) is a 5 bed unit, which admits ∼1% of all neonatal patients/year.

### Description of neonatal unit cases

Sixteen individuals with *E. cloacae* bloodstream infection were identified in the hospital neonatal unit between 18 November 2012 and 9 February 2013. Details on ward movements, sampling and outcome were collected retrospectively. Surveillance incorporated any cases observed in the clean and septic nurseries, the NICU and the paediatric ICU (PICU). Any further blood culture isolates (i.e. serially sampled isolates from single individuals) identified during the same admission were also studied. In addition, two isolates from adult patients admitted to the emergency room during the same period with community-associated, drug-resistant *E. cloacae*-positive infections (sample taken ≤48 h prior to hospital admission) were also sequenced, on the basis that they had atypical susceptibility patterns by disc diffusion (one colistin resistant and one carbapenem and colistin resistant). Environmental sampling, which is performed fortnightly before and after intensive cleaning from five different sites in the neonatal unit,^15^ yielded an isolate from a swab taken from inside the soap container by the NICU sink.

### Microbiological methods

Clinical samples were processed locally following standard protocols. Antimicrobial susceptibility testing was performed using disc diffusion^16^ and retested in Oxford using the BD Phoenix Automated Microbiology System (BD, Oxford, UK)^17^ following the manufacturer's instructions. Species identification was based on biochemical profiling. Isolates were subcultured and sent to Oxford for long-term storage at −80°C and subsequent DNA extraction and sequencing.

### DNA extraction and sequencing

DNA was extracted from subcultured frozen stocks of isolates using a commercial kit [QuickGene (Fujifilm, Minato, Tokyo, Japan) as per the manufacturer's instructions, with an additional mechanical lysis step (FastPrep, MP Biomedicals, Santa Ana, CA, USA)]. All extracts were sequenced on the HiSeq 2500 platform (Illumina, San Diego, CA, USA), generating 151 base paired-end reads.

We also used sequence data from 14 *E. cloacae* cases sampled between January 2008 and May 2012 thought to be unrelated to the set of cases under investigation for this study and part of a separate study (NCBI project number: PRJNA253300).^15^ These were from a random sample of stored Enterobacteriaceae isolates processed in the hospital microbiology laboratory, stratified by susceptibility profile, age (adults/children) and hospital- (sampled ≥48 h after admission) versus community-associated infections (sampled <48 h after admission, no previous admission to our hospital and delivery outside of a healthcare facility).

### Sequence data processing and analysis

Illumina reads were mapped to a number of publicly available *bla*_NDM_-containing plasmid reference sequences (GenBank accession numbers AB759690.1, AP012055.1, AP012208.1, HG003695.1, HQ451074.1, JN377410.2, JN420336.1, JQ314407.1, JX182975.1 and KF295828.1) using the Burrows–Wheeler Aligner^18^ and coverage visualized using in-house R scripts. Only pKOX_NDM1 (RefSeq: NC_021501.1; 110 781 bp) showed substantial mapping, with nearly all of the reference mapped; for the others, <20% of the reference was mapped in each case. Reads were, therefore, subsequently remapped to a pseudoreference consisting of the *E. cloacae* chromosomal reference NCTC 9394 (RefSeq: NC_021046.1; 4 908 759 bp) and the pKOX_NDM1 reference, with mapping and base calling performed using the methods described previously;^19^ these data were used in all relevant downstream analyses.

For *Citrobacter* isolates, reads were mapped to the *Citrobacter freundii* 4_7_47CFAA reference (RefSeq: GCF_000238735.1; 5 122 674 bp) as above.^19^

We used Velvet and VelvetOptimiser for *de novo* assembly.^20,21^ Presence/absence of resistance gene variants encoding β-lactam, quinolone and aminoglycoside resistance in assemblies was determined using BLASTn.^22^ *In silico* typing of plasmid replicons was done by comparing the relevant reference coding sequences from the PlasmidFinder online database (http://cge.cbs.dtu.dk/services/PlasmidFinder/)^23^ with *de novo* assembled contigs using BLASTn; specific replicons were considered as present using the same identity cut-off (95%) specified as the default by PlasmidFinder (i.e. a minimum of 95% of the nucleotides in the reference replicon have a match in the corresponding sequence identified in the *de novo* assembly). ST determined by MLST was assigned using a similar method of comparison with reference alleles and profiles in the Miyoshi-Akiyama scheme^24^ (results are presented in Table S1, available as Supplementary data at *JAC* Online).

Presence/absence of pKOX_NDM1 was defined on the basis of BLASTn hits against each isolate's *de novo* assembly. Presence in each 1000 bp window of the reference sequence was defined as ≥90% of that region being present in one or more blast hits, after filtering out hits that were <200 bp or showed <95% sequence identity.

Phylogenetic relationships between strains on the basis of core chromosomal, single nucleotide variants (SNVs; core SNVs defined as no null call in any isolate at that site) were inferred under a maximum-likelihood model in PhyML using settings described previously.^15^

*De novo* assembled contiguous sequences (contigs) were annotated using Prokka.^25^ Annotated coding sequences were clustered at a 90% protein threshold using CD-HIT^26^ to establish a protein pan-genome for the whole dataset; representative sequences for each cluster were then set up as a blast database to query presence/absence across the dataset protein pan-genome using tBLASTn. The core genome was defined as the subset of coding sequences present in all isolates; the accessory genome was defined as representing coding sequences absent in at least one of the isolates. Presence was defined as a relative coverage of a coding sequence of >80%, with relative coverage defined as percentage sequence homology × percentage match length. Presence/absence was depicted using the heatmap.2 package in R.

Sequence data for the strains sequenced from this study are deposited with the NCBI under project number PRJNA263245. Strain details, including a summary of sequencing, mapping and *de novo* assembly statistics for each isolate, are listed in Table S1.

## Results

**Figure 1.**
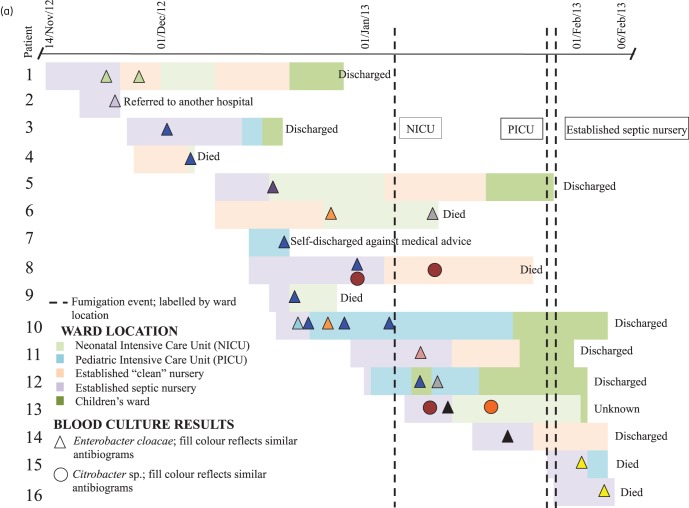

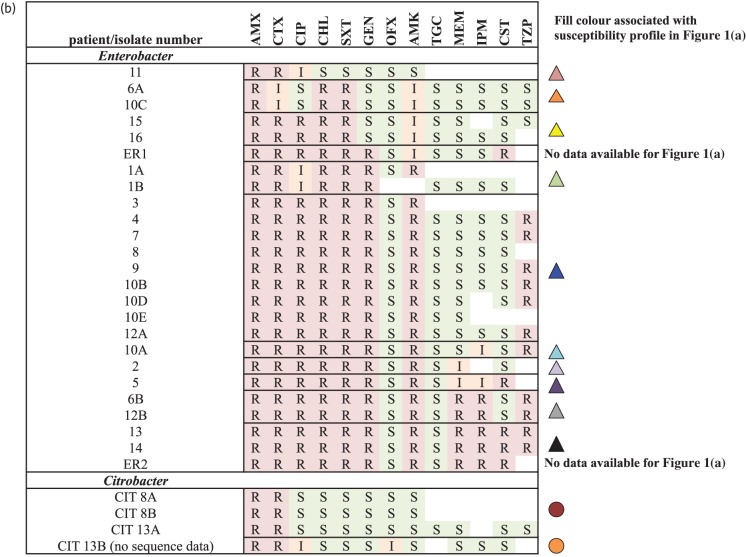
Timeline of the outbreak, with each set of horizontal bars representing a patient admission, coloured by ward location, and annotated with culture result and outcome. (b) Disc diffusion-based phenotypic susceptibility profiles from the hospital laboratory for isolates represented in (a). Fill colours of the isolates in (a) reflect clustering on the basis of disc diffusion-based phenotypic profiles in (b). Clustering is based on the most likely susceptibility profile given incomplete data. Epidemiological data for ‘ER1’ and ‘ER2’ were not available. Broken lines represent the occurrence of fumigation events in single units. AMX, amoxicillin; CTX, cefotaxime; CIP, ciprofloxacin; CHL, chloramphenicol; SXT, trimethoprim/sulfamethoxazole; GEN, gentamicin; OFX, ofloxacin; AMK, amikacin; TGC, tigecycline; MEM, meropenem; IPM, imipenem; CST, colistin; TZP, piperacillin/tazobactam.

For the 16 neonates with *E. cloacae* bloodstream infection, the median time from admission (day of delivery in all cases) to first positive blood culture was 6.5 days (IQR: 4–8 days). Three neonates had *E. cloacae*-positive urine cultures (two on the same day as the blood culture and one a day later); one had a positive CSF culture 6 days later (none of these isolates was available for sequencing). Two patients were coinfected with *Citrobacter* spp. Six neonates died (46% of the cases where the outcome was known; ultimate outcome was unclear in three cases). Figure 1(a) shows the epidemiological timeline of infected neonatal cases. Fumigation with an Ecoshield system [hydrogen peroxide (H_2_O_2_) at 20%] was used to try to limit transmission (Figure 1a); after three of the affected units (NICU, PICU and the septic nursery) were fumigated, only two further cases were observed (Patients 15 and 16).

Sequence data were available for 43 isolates in total, including: (i) 16 original *E. cloacae* isolates in the neonatal unit case cluster; (ii) 7 longitudinal *E. cloacae*-positive isolates from neonates in the cluster who were persistently bacteraemic (four patients); (iii) 2 community-associated drug-resistant *E. cloacae* strains sampled from adults attending the emergency room; (iv) the historical *E. cloacae* invasive isolates from invasive infections (11 hospital-associated and 3 community-associated); and (iv) 3 *Citrobacter* spp. blood culture isolates obtained from neonates in the unit case cluster concomitantly infected with *E. cloacae*. Sequence data were not successfully obtained for one *Citrobacter* sp. isolate (CIT 13B; second isolate in Patient 13).

**Figure 2.**
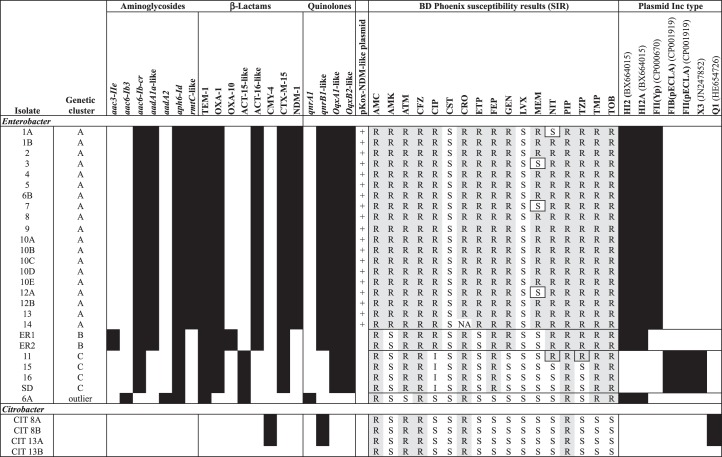
Resistance gene presence (black shading)/absence (white shading) for each isolate using genomic data and corresponding antimicrobial susceptibility profiling using the BD Phoenix Automated System (automated broth microdilution method). Black borders have been placed around individual susceptibility results that represent a genotype/phenotype discrepancy. Black borders around global susceptibility profiles correspond to host strain genetic clusters. Plasmid Inc types identified in isolates are represented by black shading. Sequence data were not available for CIT 13B. AMC, amoxicillin/clavulanate; AMK, amikacin; ATM, aztreonam; CFZ, cefazolin; CIP, ciprofloxacin; CST, colistin; CRO, ceftriaxone; ETP, ertapenem; FEP, cefepime; GEN, gentamicin; LVX, levofloxacin; MEM, meropenem; NIT, nitrofurantoin; PIP, piperacillin; TZP, piperacillin/tazobactam; TMP, trimethoprim; TOB, tobramycin; SIR, susceptible, intermediate or resistant, respectively.

**Figure 3.**
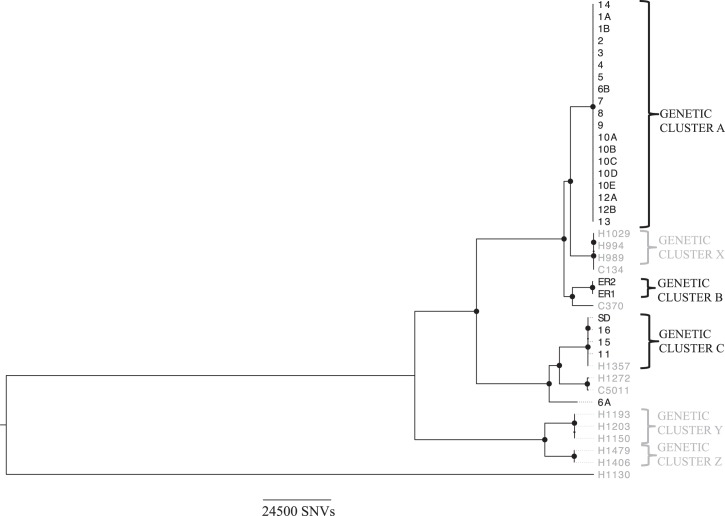
Maximum-likelihood phylogeny of the study isolates, including sequence data from a set of previously sequenced historical *E. cloacae* isolates (in grey). Closely related isolates (<10 SNVs apart) are labelled with brackets and defined as Genetic clusters A–C and X–Z. Large circles represent nodes supported by bootstrap values of >95%. An ‘H’ prefix denotes a hospital-associated isolate, a ‘C’ prefix denotes a community-associated isolate and ‘SD’ represents the soap dispenser isolate; all other isolates were from clinical samples. H1130 represents a genetically divergent outlier within the *E. cloacae* group, with >309 000 SNVs between it and Cluster A isolates.

The isolates causing infection showed large variation in antimicrobial susceptibilities based on locally available disc diffusion results (Figure 1b), with 10 distinct *E. cloacae* susceptibility profiles identified in the 16 neonates with epidemiological information available (plus 1 additional profile in an adult without epidemiological data) and 2 *Citrobacter* profiles, complicating the definition of epidemiological case clusters. Broth microdilution results were more consistent between isolates, showing seven distinct *E. cloacae* profiles and one *Citrobacter* profile (Figure 2). In contrast, gene presence/absence identified only four *E. cloacae* profiles, with five isolates showing discrepancies with regard to broth microdilution results (Figure 2). Resistance gene presence corresponded fully with bacterial strain genetic clusters (Figures 2 and 3; genetic clusters contained strains with <10 SNVs). Nineteen isolates from 13 individuals (all isolates in Genetic cluster A) were *bla*_NDM-1_ positive; 3 of these were susceptible to meropenem by broth microdilution, while 11/17 tested (65%) had been susceptible by disc diffusion.

Across all cases and locally sampled but historical isolates, a mean of 82% (range: 71%–87%) of the NCTC 9394 reference genome was called and 399 874 core SNVs identified, with several distinct closely related genetic clusters (<10 SNVs apart). Amongst the isolates sampled for the present study, based on the core chromosome, there were three of these genetic clusters (Figure 3, Clusters A–C) and a single outlier (Isolate 6A). Cluster A consisted of 19 of the 23 neonatal isolates; Cluster B included only the 2 adult isolates sampled in the emergency room; and Cluster C consisted of 3 neonatal isolates, as well as the isolate from the soap dispenser and a historical isolate (H1357), isolated 3 February 2012 (Figure 3). The genetic distance between clusters was substantial (>17 000 SNVs between A and B and >68 000 SNVs between A or B and C). Within clusters, there were two SNVs amongst isolates in Cluster A (one in Isolate 7 only and one in Isolate 8 only), no SNVs in Cluster B and six in Cluster C, all of which were found exclusively in the historical isolate H1357. Notably, the isolate from the soap dispenser (Isolate SD) was 0 SNVs apart from the neonatal isolates in that genetic cluster, supporting its role as a potential source of transmission for the other three temporally associated isolates within Cluster C.

For the three *Citrobacter* isolates for which sequencing data were available, 79%–80% of the 4_7_47CFAA reference was called in each isolate. There were only two SNVs separating the three isolates, indicating very low genetic variation.

The pan-genome for all cases and historical isolates, including *Citrobacter* sp., contained 14 154 representative coding sequences, of which 11 448 were found in at least one *Enterobacter* isolate. Of these, 4566 were considered core and 6882 accessory; here there was some evidence of shared subsets of the accessory genome across genetic clusters and minimal variation within these clusters (Figure S1).

Plasmid replicon types present in the isolates included: (i) in Cluster A, IncHI2 and HI2A, both located on the same contig in all isolates, and IncFII(Yp); (ii) in Cluster B, IncHI2 and HI2A, both located on the same contig in all isolates; (iii) in Cluster C, FIB(pECLA) and FII(pECLA), both located on the same contig in all isolates, and X3; (iv) in Isolate 6A, IncHI2 and HI2A, both located on the same contig; and (v) in the *Citrobacter* isolates, Q1 only (Figure 2).

NDM-1 was present in all Cluster A isolates and none of the other contemporary neonatal or ER isolates. Mapping of reads to publicly available NDM-containing plasmid references demonstrated only limited evidence for the presence of these plasmids in any isolates, except for the IncFII plasmid pKOX_NDM1 (RefSeq: NC_021501.1), where all but a 13 kb region was present in the Cluster A isolates (Figure S2), and the other contemporary isolates showed very minimal homology. One of the historical community-associated isolates (C370; known to be NDM-1 positive) from January 2009 also showed high similarity to pKOX_NDM1, suggesting that a pKOX_NDM1-like plasmid may have been in circulation for ≥3 years. No core nucleotide-level variation in pKOX_NDM1 sequence was observed amongst the NDM-1-positive isolates.

## Discussion

Our WGS analysis provided strong evidence for the existence of a number of transmission networks involving *E. cloacae* in this context, in contrast to the prevailing belief that *E. cloacae* infections are typically caused by either discrete invasion events in single human hosts or sporadic, clonal outbreaks of disease.^4^ Two distinct neonatal *E. cloacae* outbreaks were identified in the neonatal unit during the study period, contemporaneous with a separate group of drug-resistant community-associated adult cases sampled in the emergency room (Figure 3; Cluster B). In addition, three historical hospital-associated genetic clusters were observed (Figure 3; Clusters X, Y and Z). Sequencing of three *Citrobacter* isolates from two individuals revealed minimal genomic variation (two SNVs). As the *E. cloacae* isolates from these individuals both fell into Cluster A, this suggests that there may have been concurrent transmission of both species.

The largest neonatal outbreak was associated with an NDM-1 plasmid showing substantial homology to pKOX_NDM1. The second, smaller, neonatal outbreak was potentially attributable to the contamination of a soap dispenser on the unit. The NDM-1 outbreak was clonal at the level of the host strain and the NDM-1 resistance plasmid, with only two chromosomal and no plasmid SNVs. In addition, the genetic homogeneity demonstrated in the accessory genome within the outbreak suggested that the majority of other chromosomal accessory/episomal components were also shared.

Discrepancies between straightforward phenotyping methods and the presence of resistance genes are well described^22,27^ and may reflect a range of issues, including: the consistency of phenotyping across methods; known difficulties in assessing susceptibility for particular antimicrobials with some methods, such as piperacillin/tazobactam; the significance of a susceptible/resistant cut-off for concentrations close to the breakpoint; variability in gene expression; and loss of resistance genes in storage/repeat culture. For both susceptibility phenotyping and identification of transmission, this study demonstrates that the genetic determination of resistance mechanisms proved the most consistent method compared with either disc diffusion methods or BD Phoenix and is particularly helpful in the context of widespread antimicrobial resistance in organisms capable of carrying a number of different resistance mechanisms encoding the same phenotype. The low genetic diversity observed in core and accessory genomes within Cluster A makes the phenotypic variability in susceptibility profiles more likely to be due to environmental factors.

Refillable soap dispensers that do not represent a closed system are a recognized reservoir for Enterobacteriaceae^28^ and have been implicated as sources of pathogens causing nosocomial outbreaks.^29,30^ This is of particular concern given that handwashing is recognized as a cornerstone of standard infection control practice and is being promoted across resource-limited settings. In this healthcare unit, as in many resource-limited settings, soap containers are of the refillable variety and could act as a sporadic reservoir for the transmission of disease-causing isolates, including drug-resistant strains.

Fumigation with H_2_O_2_ was used in an attempt to limit the emergence of further cases of infection and was carried out during the outbreaks on three of the four major paediatric critical care units involved. In this situation, fumigation of all units may have eventually contributed to the termination of the NDM-1-associated outbreak, but had limited effect on the possible soap dispenser-associated outbreak, with genetically linked cases occurring before and after the three fumigation events. This is plausible given that soap contained within a soap dispenser would be unlikely to be affected by fumigation. The limited impact of the first fumigation event on subsequent transmission could be explained by a number of reasons: fumigation was not carried out simultaneously in all units and fumigated units may have been recolonized from untreated units; other patients or healthcare workers may have been acting as reservoirs for strains involved in the outbreak; or it may be that fumigation has less of an impact on *Enterobacter* strains, despite the fact that it has been shown to be effective in limiting outbreaks caused by several healthcare-associated pathogens in a recent systematic review.^31^

Repetitive sequences prevent accurate assembly from short-read sequence data. As these are often present in drug resistance plasmids, the resulting structures can be difficult to resolve. One study limitation is that with the short-read Illumina sequences generated, we were not able to fully describe the plasmid structures present in each of the isolates; this would have required a separate, long-read sequencing approach. However, the wider accessibility and decreasing cost of sequencing mean that the publicly available set of plasmid reference sequences is growing rapidly and in this case enabled us to identify a *bla*_NDM-1_-containing plasmid closely related to one of these reference sequences, namely pKOX_NDM1.

As a result of access to a set of historical *E. cloacae* sequence data from the same locality, we were also able to place the outbreak isolates in a wider genetic context. This demonstrated a genetic link between one of the neonatal clusters (Cluster C) and a clinical, hospital-associated isolate sampled a year previously, suggesting that this may represent a hospital-associated clone that has caused at least two temporal clusters of cases. In addition, we identified an episomal link involving an NDM-1 plasmid present in another cluster (Cluster A) and a community-associated isolate with a highly divergent chromosomal background (>18 000 SNVs) from 3 years earlier. This, and the fact that pKOX_NDM1 was first identified in a *Klebsiella oxytoca* isolate from an individual hospitalized in Taiwan in November 2010,^32^ demonstrates the wide sampling frames with respect to timeframe, species and geography that will be required to fully understand the dynamics of NDM-1 plasmid transmission and likely other resistance gene plasmids in Enterobacteriaceae.

## Funding

This work was supported by the Health Innovation Challenge Fund (grant: HICF-T5-358), a parallel funding partnership between the Department of Health and Wellcome Trust, the National Institute for Health Research (NIHR) Oxford Biomedical Research Centre and the UKCRC Modernising Medical Microbiology Consortium, with the latter being funded under the UKCRC Translational Infection Research Initiative supported by the Medical Research Council, the Biotechnology and Biological Sciences Research Council and the NIHR on behalf of the Department of Health (Grant G0800778) and the Wellcome Trust (Grant 087646/Z/08/Z). N. S. is a clinical research fellow funded by the Wellcome Trust.

## Transparency declarations

None to declare.

## Disclaimer

The views expressed are those of the authors and not necessarily those of the National Health Service, the NIHR, the Department of Health or the Wellcome Trust.

## Supplementary data

Table S1, Figure S1 and Figure S2 are available as Supplementary data at *JAC* Online (http://jac.oxfordjournals.org/).

Supplementary Data
