# Supplementary material for: Dynamics of MDR Enterobacter cloacae outbreaks in a neonatal unit in Nepal: insights using wider sampling frames and next-generation sequencing
Source: J Antimicrob Chemother. 2015 Jan 3;70(4):1008–15. doi: 10.1093/jac/dku521 (PMC4356206; doi:10.1093/jac/dku521)
Supplement: Supplementary Data [file supp_70_4_1008__index.html]

Dynamics of MDR Enterobacter cloacae outbreaks in a neonatal unit in Nepal: insights using wider sampling frames and next-generation sequencing — Supplementary Data 

# Dynamics of MDR *Enterobacter cloacae* outbreaks in a neonatal unit in Nepal: insights using wider sampling frames and next-generation sequencing

## Supplementary Data

Supplementary Data

**Files in this Data Supplement:**

- Supplementary Figures - doc file
- Supplementary Table 1 - xlsx file
