# Supplementary material for: Dynamics of MDR Enterobacter cloacae outbreaks in a neonatal unit in Nepal: insights using wider sampling frames and next-generation sequencing
Source: J Antimicrob Chemother. 2015 Jan 3;70(4):1008–15. doi: 10.1093/jac/dku521 (PMC4356206; doi:10.1093/jac/dku521)
Supplement: Supplementary Data [file supp_dku521_dku521supp_figs.doc]

**Figure S1. Presence/absence of non-redundant coding sequences of the dataset-derived accessory genome in each of the study isolates.** Each vertical column represents a coding sequence. Deep purple denotes presence at >90%, paler purple at 80-90%, and white <80% relative coverage. Isolates are ordered to be consistent with the core phylogeny in main text Fig.3.

**Figure S2. Comparisons of study isolates with reference plasmid pKOX_NDM1.** Purple indicates presence, and white indicates absence, for each 1000 bp window. The scale indicates position along the pKOX_NDM1 reference, in kb. The position of *bla*_NDM-1_ in the reference (38689-39501 [complement]) is marked with an arrow. Isolates are ordered to be consistent with the core phylogeny in main text Fig.3. The small “deletion” observed in isolate 10A with respect to the reference represents a 117 bp region that is absent in the *de novo* assembly, coinciding with a contig breakpoint. Investigation of the mapped data showed no support for a deletion in this region and it is therefore likely to be an artefact of the *de novo* assembly.
